# Protein Kinase CK2 Expression Predicts Relapse Survival in ERα Dependent Breast Cancer, and Modulates ERα Expression *in Vitro*

**DOI:** 10.3390/ijerph13010036

**Published:** 2015-12-22

**Authors:** Marlon D. Williams, Thu Nguyen, Patrick P. Carriere, Syreeta L. Tilghman, Christopher Williams

**Affiliations:** 1 College of Pharmacy, Xavier University of Louisiana, 1 Drexel Dr, New Orleans, LA 70125; mwilli41@xula.edu (M.D.W.); tnguye71@xula.edu (T.N.); pcarriere@msm.edu (P.P.C.); 2 Division of Basic Sciences, College of Pharmacy and Pharmaceutical Sciences, Florida Agricultural & Mechanical University, 1415 S. Martin L. King Jr. Blvd., Tallahassee, FL 32307; syreeta.tilghman@famu.edu

**Keywords:** CK2, estrogen receptor (ERα), relapse free survival, breast cancer

## Abstract

The heterotetrameric protein kinase CK2 has been associated with oncogenic transformation, and our previous studies have shown that it may affect estrogenic signaling. Here, we investigate the role of the protein kinase CK2 in regulating ERα (estrogen receptor α) signaling in breast cancer. We determined the correlation of CK2α expression with relapse free breast cancer patient survival utilizing Kaplan Meier Plotter (kmplot.com/analysis/) to mine breast cancer microarrays repositories. Patients were stratified according to ERα status, histological grade, and hormonal therapy. Luciferase reporter assays and flow cytometry were implemented to determine the impact of CK2 inhibition on ERE-mediated gene expression and expression of ERα protein. CK2α expression is associated with shorter relapse free survival among ERα (+) patients with grade 1 or 2 tumors, as well as among those patients receiving hormonal therapy. Biochemical inhibition of CK2 activity results in increased ER-transactivation as well as increased expression among ERα (+) and ERα (−) breast cancer cell lines. These findings suggest that CK2 may contribute to estrogen-independent cell proliferation and breast tumor progression, and may potentially serve as a biomarker and pharmacological target in breast cancer.

## 1. Introduction

Despite recent advances in detection and therapeutic intervention, breast cancer remains the second most common cause of cancer-related death among American women. Most breast cancers are ERα (+), making those patients eligible for treatment with hormonal therapy. However, as many as 60% of women progress to become resistant to hormonal therapies [1]. Furthermore, a minority of patients have ERα (−) disease, and are inherently resistant to hormonal therapies. In either case, patients are relegated to cytotoxic chemotherapy and are subject to the associated severe side effects. As such, it is imperative to determine the mechanisms by which patients develop estrogen resistance, as well as to develop more targeted therapies to reverse endocrine resistance in breast cancer.

Protein kinase CK2 is a pleiotropic, heterotetrameric serine kinase consisting of two regulatory β subunits, and 2 catalytic subunits, (α and α’) [2,3]. CK2 activity has been shown to be significantly elevated in cancerous versus normal tissues, including patient-matched colorectal and breast tissues [4]. As such, several studies have been initiated to identify drugs which selectively and potently inhibit CK2 activity [5]. Two such agents CIGB-300 (cyclic peptide) and silmitasertib (CX-4945) are currently being targeted in two clinical trials for the treatment of cervical cancer (CIGB-300), or multiple myeloma, cholangiosarcoma, and advanced solid tumors including breast (CX-4945) [6,7]. More recently, a large scale *in vitro* screening of the NIH/NCI chemical Diversity Set Library has identified 1,3-Dichloro-6-[(E)-((4-methoxyphenyl)imino)methyl] dibenzo(b,d) furan-2,7-diol as a novel potent, selective and cell permeable inhibitor of protein kinase CK2 [8]. Additionally, molecular modeling studies have led to the development of a series of dihydroxyindeno [1,2-*b*]indole derivatives which inhibit CK2 kinase activity at nanomloar concentrations [9]. These studies highlight the potential for development of pharmacological inhibitors of CK2 for cancer treatment.

A recent study by Ortega *et al*., showed that CK2α mRNA is overexpressed in cancerous *versus* normal tissue of the breast, lung, ovarian, prostate, renal, and colon, as well as others [10]. Another recent study has demonstrated that CK2α is statistically overexpressed in basal breast cancer as compared to either normal breast epithelium or the well-differentiated luminal a subtype [11]. In tumor microarray studies, CK2α was identified as a part of a 9-marker gene expression signature associated with increased metastatic risk among breast cancer patients [6]. Studies have shown that transgenic overexpression of CK2α in the rat mammary gland results in tumor development in 30% of animals [12]. Additionally, it was shown that 20 out of 21 independent gene expression microarrays tested, CK2α was overexpressed in breast carcinoma versus normal breast epithelium [10]. Together, these studies suggest that overexpression of CK2 may be causative in human breast oncogenesis. Interestingly, *in vitro* studies have shown that DMAT, the selective inhibitor of CK2, induces apoptosis in tamoxifen-resistant, but not wild-type MCF-7 breast cancer cell lines [13]. Our own previously published studies have shown that CK2 can phosphorylate human ERα at S282 and S559 both *in vitro* and *in vivo*, and that mutation of CK2 sites results in a moderate but significant increase in ERα transcriptional activity [14].

Where most findings have suggested that CK2 is a negative prognostic indicator in breast cancer, no studies to date have examined the intersection between ERα and CK2α signaling. Here, we mined publically available microarray repositories to ascertain the significance of CK2α expression to relapse free survival (RFS) based on ERα status, histological grade, and hormonal therapy. We investigated the impact of CK2 inhibition on ER-dependent transactivation *in vitro*, and subsequently of ER expression. Our findings suggest that CK2 is a key signaling component in the progression of ER (+) breast cancer to a more aggressive phenotype.

## 2. Materials and Methods

### 2.1. Kaplan Meier Survival Analysis

The application of KM plot has been described in detail previously [15,16]. Briefly, Kaplan Meier (KM) plots were attained using the KMPlotter web-based (kmplot.com/analysis) curator, which surveys public microarray repositories for relapse free and overall survival among patients with breast, lung, ovarian or gastric cancers. The KMplotter recognizes 54,675 individual Affymetrix probesets, and surveys expression data from 4142 breast cancer patients (as of 2014). Survival and gene expression data were derived from the GEO (Gene Expression Omnibus), TCGA (The Cancer Genome Atlas), and EGA (European Genome-phenome Atlas) databases. In our analysis, patients were differentiated based on ERα expression, histological tumor grade, or as recipients of hormonal therapy. In order to ascertain CK2α expression, Affymetrix probe 203575 was selected, which corresponds to both CK2α (CSNK2A1) and CK2α’ (CNK2A2). Populations were split by median CK2α expression and plots generated accordingly.

### 2.2. Cell Culture

T47D and MDA-MB-231 cells were obtained from the American Tissue Type Collection (ATTC). Stably transfected T47D-luciferase cells were a gift from the Xavier University of Louisiana RCMI Cell and Molecular Biology core facility. All cell lines were maintained in phenol red free RPMI 1640 media supplemented with 10% fetal bovine serum (FBS) and penicillin-streptomycin. Cells were sub-cultivated twice weekly.

### 2.3. Luciferase Reporter Assay

Stably transfected T47D-ERE-luciferase cells were cultured in phenol red free RPMI 1640 media supplemented with 10% charcoal-dextran treated FBS (Life Technologies, Grand Island, NY USA) for a minimum of 72 h. Subsequently, cells were incubated with TBCA (tetra-bromo-cinnamic acid EMD Millipore, Billerica, MA USA) and/or fulvestrant (Santa Cruz Biotechnologies, Dallas, TX, USA) for 24 h. Cells were then lysed and luciferase activity was assayed using Bright-Glo^©^ (Promega Madison, WI, USA) detection system. Experiments were performed three times in triplicate per experimental repeat.

### 2.4. Flow Cytometric Detection of ERα

We detected ERα protein levels using flow cytometry as described previously [17]. T47D and MDA-MB-231 cells were cultured in phenol red free RPMI 1640 media supplemented with 10% charcoal-dextran treated FBS for a minimum of 72 h. Subsequently, cells were incubated with TBCA for 24 h and afterwards cells were collected by trypsinization and fixed in 10% formalin. The fixed cells were then blocked and permeabilized in a blocking buffer (phosphate buffered saline, 10% goat serum, 0.1% Nonidet P-40). Samples were then incubated in blocking buffer and anti-ERα antibody (HC-20, Santa Cruz Biotechnology Dallas, TX, USA) at 1:50 dilution and incubated overnight at 4 °C. Controls received no primary antibody (negative control). After 3 washes, all samples were incubated for 1 h with AlexaFluor 647 goat α rabbit secondary antibody (1:1000). Samples were analyzed on an Accuri C6 flow cytometer, and nonspecific staining was set with the negative control. The experiment was performed with a minimum of 3 repeats.

## 3. Results and Discussion

### 3.1. CK2α Overexpression is Associated with Decreased RFS in Patients with ERα (+) Breast Cancer

**Figure 1:**
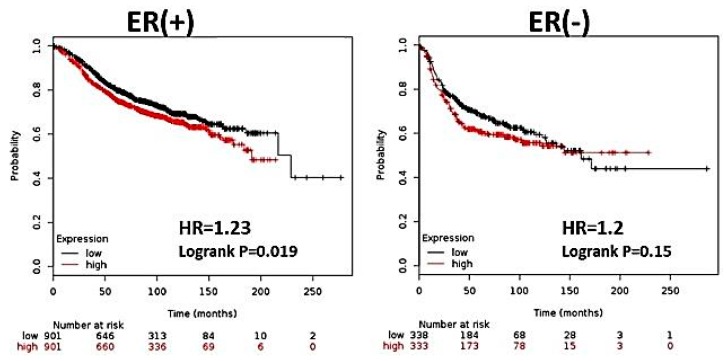
CK2α overexpression is associated with decreased RFS in patients with ER (+) breast cancer. Using KMPlotter, we interrogated publically available microarray repositories for breast cancer to determine if CK2α expression was associated with different survival rates among ERα (+) and ERα (−) breast cancer patients. Hazard ratio (HR) and Logrank P values are shown. Low expression of CK2α expression (below median) is noted in black, and the high CK2α expression (above median) is noted in red.

Previous studies have shown an overall decrease in RFS among patients with high expression of CK2α [16]. However, no studies have stratified patients according to ER status to determine if there are differences in patient survival among those with ERα (+) or ERα (−) tumors. We selected patient profiles based on ERα status, and ascertained RFS based on CK2α expression (below median = low, above median = high). We observed that high CK2α expression is associated with a moderate but significant increase in risk of relapse among ERα (+), but not ER (−) breast cancer patients (*p* = 0.019 and *p* = 0.15, respectively) (Figure 1). It is notable that even in ER (−) tumors, high CK2α expression trended toward shorter RFS, though this was not significant. These findings are in agreement with other findings showing that overall, CK2 is a negative prognostic indicator in cancer and that multiple mechanisms may be associated with its oncogenic potential.

**Figure 2:**
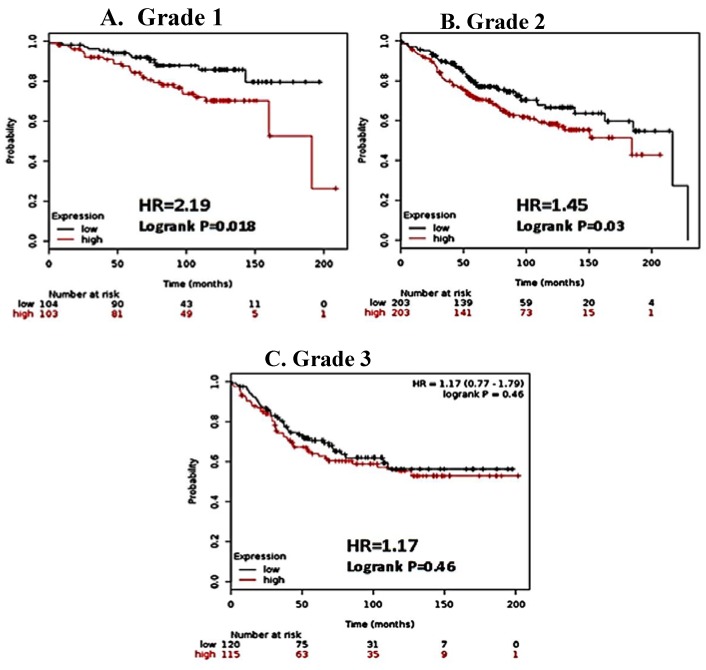
CK2α expression is most strongly associated with shorter RFS in lower grade breast cancer. Km Plotter was used to stratify patient microarray data by histological grade. Hazard ratio (HR) and Logrank P values are shown. Low expression of CK2α expression (below median) is noted in black, and the high CK2α expression (above median) is noted in red. (**A**) and (**B**), expression of CKα correlated with decreased relapse free survival patients with grade 1 and grade 2 cancer, respectively. (**C**) CK2α expression was not correlated in patients with grade 3 cancer.

**Figure 3:**
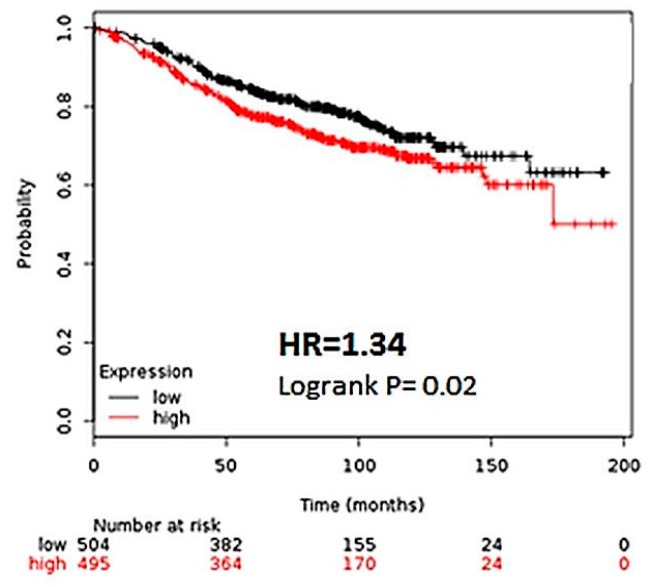
The CK2α expression is predictive of relapse in patients on hormonal therapy. In Km Plotter, microarray data was selected from patients that had received hormonal therapy during treatment for breast cancer. The data was then stratified according to CK2α expression. Hazard ratio (HR) and Logrank *p* values are shown. Low expression of CK2α expression (below median) is noted in black, and the high CK2α expression (above median) is noted in red.

Interestingly, a more specific effect can be observed when ER (+) patients are further stratified by histological grade. Among patients with grade 1 cancer, a greater than 2 fold increase in recurrence risk (HR 2.19, CI) was observed among those with high CK2α expression (Figure 2A). However, high CK2α expression shows less of a correlation with RFS in more advanced cancers, with a 45% increase in risk among patients with grade 2 cancers and no statistical difference among patients with grade 3 cancers (Figure 2B,C). This suggests that early in the etiology of ER (+) breast cancer, CK2α plays a significant role in disease progression. The gradual loss of this effect could be due to parallel oncogenic pathways being activated in which the influence of CK2 is less pronounced. For most patients with ER (+) breast cancer, hormonal therapy designed to abrogate estrogen dependent cell proliferation is implemented [18]. Since the patient population for which the greatest difference was observed were those with ER (+) disease, we sought to determine if CK2α expression levels correlated to survival rates of patients undergoing hormonal therapy. As such, we selected patients who had undergone hormonal therapy for breast cancer, and secondarily stratified the patients based on CK2α expression, as described previously. Significantly, patients with a lower CK2α expression enjoyed longer relapse-free survival than those patients with high CK2α expression, with 34% decrease in risk of recurrence (Figure 3). This supports our contention that CK2α contributes to the development of hormone resistance in ER (+) breast cancer.

### 3.2. Inhibition of CK2α Results in Increased ER Transactivation in Stably Transfected T47D-luciferase Cells

**Figure 4:**
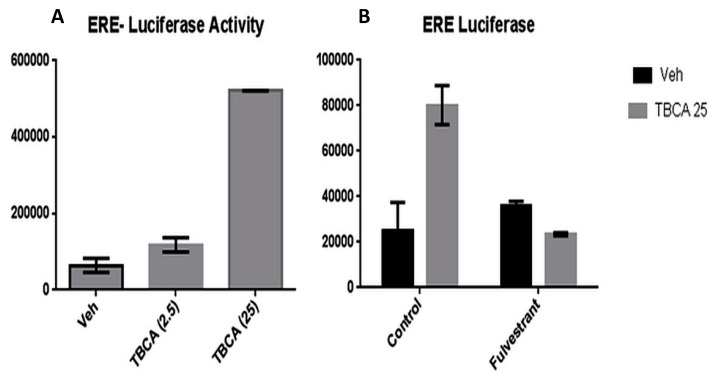
CK2 inhibition results in increased ERα-dependent ERE-transcriptional activity. T47D cells stably transfected with an ERE-luciferase were incubated in the presence of (**A**) 2.5 or 25 μM TBCA or (**B**) fulvestrant (1 μM) and/or TBCA (25 μM) for 24 h. Luciferase activity was measured was ascertained after 24 h. Each study was performed for at least 3 experimental repeats.

Our previous studies have shown that CK2α phosphorylated ERα at S282 and S559. Serine to alanine mutation of these sites results in a moderate increase in ERE driven gene expression, suggesting that CK2 has a repressive effect of ERα transcription activity [19]. Here, we investigated the ability of tetrabromocinnamic acid, a selective inhibitor of CK2 kinase activity, to impact transcriptional activity in T47D cells stably expressing an ERE-driven luciferase reporter [20]. After 24 h, TBCA treatment resulted in a significant increase in ERE-luciferase activity (Figure 4A). To determine if the induction of ER transcriptional activity was due to an effect on ERα as opposed to other components of the transcriptional machinery, we treated the cells with fulvestrant, a pure ERα antagonist that causes degradation of the ERα [21]. Fulvestrant inhibited TBCA-induced transcriptional activity, confirming that the observed effect of TBCA on luciferase activity was mediated through ERα (Figure 4B). These studies provide further evidence that CK2 has a negative regulatory effect on ER transcriptional activity, which might be a key adaptation for breast tumor progression in ER (+) disease.

### 3.3. TBCA Causes Increased Expression of ERα in ERα (+) Breast Cancer Cells, As Well As a Sub-Population of ERα (−) Breast Cancer Cells

**Figure 5:**
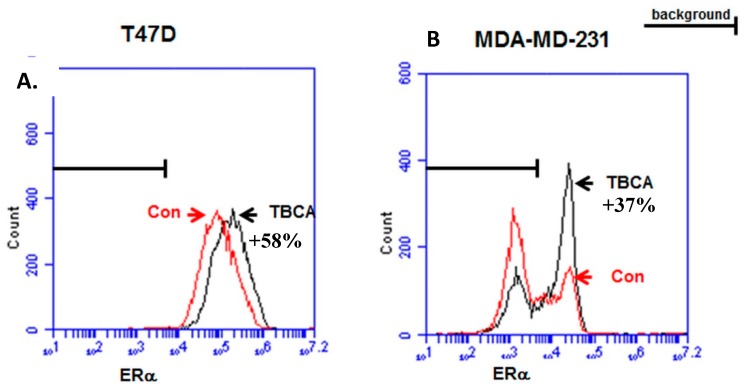
CK2 inhibition with TBCA induces ERα expression in ER (+) T47D and ER (−) MDA-MB-231 breast cancer cells. (**A**) T47D and (**B**) MDA-MB-231 cell were incubated for 24 h in the absence (Con) or presence (TBCA) of 25 μM TBCA. Cells were collected and stained with αERα antibody and fluorescent secondary antibody. Fluorescent staining was assessed by flow cytometry. Background staining was established using secondary antibody alone. Percentage values represent the ratiometric increase in ERα associated fluorescence in the presence of TBCA.

One of the most significant prognostic indicators in breast cancer is the expression of ERα. It is our contention that ERα expression and function in breast cancer cells is at least in part related to CK2 expression. In order to determine the mechanism by which CK2 might induce ER transcriptional activity, we studied the effects of CK2 inhibition on ERα expression using fluorescence immunostaining and flow cytometry. In T47D cells, we observed a substantial 58% increase in ERα expression upon treatment with TBCA (Figure 5A). Since T47D cells express a significant amount of ERα under basal conditions, we investigated whether if MDA-MB-231 cells, which are typically regarded as ERα (−), could be coerced to express ERα by inhibiting CK2. We observed that a small subpopulation of MDA-MD-231 cells expressed ERα under basal condition, and that treatment with TBCA resulted in a greater percentage of cells expressing ERα (Figure 4B). This culminated as an increased ERα signal of 37% in the total population. This suggests that CK2 may be in part responsible for cell survival/proliferation in the absence of ERα, which could in turn lead to insensitivity to hormonal therapy in breast cancer patients.

## 4. Conclusions

Data mining of publically available gene expression microarrays showed that patients with high expression of CKα may be at increased risk of relapse, despite being diagnosed with a lower grade cancer. Those patients with high CK2α expression may also be at increased risk of relapse while on hormonal therapy*. In vitro*, we have shown that pharmacological inhibition CK2 activity results in an increase in ERE-driven gene expression as well as an increase ERα protein expression. Significantly, the latter effect was observe not only in ER (+) cells, but in ER (−) breast cancer cell lines which suggest that CK2 inhibition might be a therapeutic strategy to reverse or prevent endocrine resistance on breast cancer. The studies outlined here show that CK2α is a key regulator of ER activity in breast cancer both *in vivo* and *in vitro*. Our future studies will investigate the mechanisms by which CK2 promotes ERα independent cell growth and expression of endogenous ERα driven genes.
